# Supplementary material for: Programmable RNA Targeting Using CasRx in Flies
Source: CRISPR J. 2020 Jun 17;3(3):164–76. doi: 10.1089/crispr.2020.0018 (PMC7307691; doi:10.1089/crispr.2020.0018)
Supplement: Supplemental data [file Supp_FigS1.pdf]

Supplementary Data

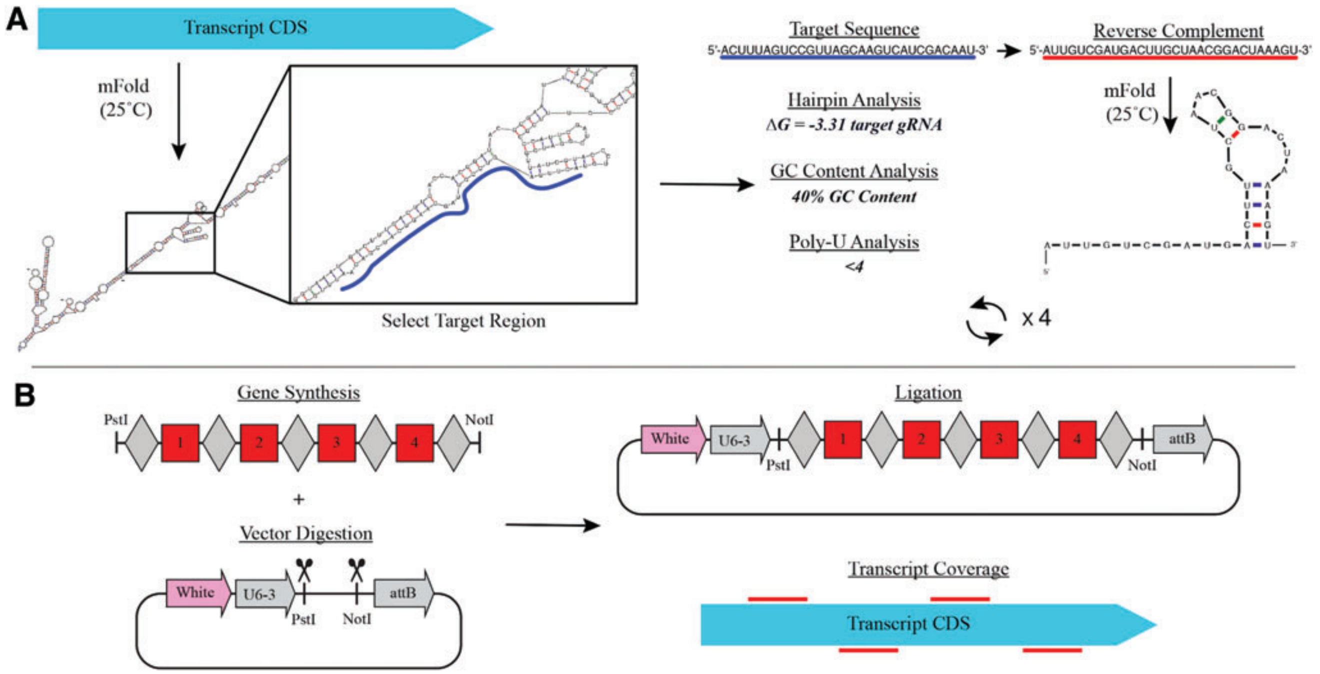

Supplementary Fig. S1. CasRx-gRNA^{array} transcript target selection and construct generation. **(A)** Schematic representing the workflow for gRNA choice. The transcript coding sequence (CDS) for a gene of interest (GOI) was entered into the mFold database (condition: 25°C) where predictive analysis identified the most probable secondary and tertiary folding of the entire transcript. We then chose specific regions that were predicted to be easily accessible for CasRx targeting (blue line), contained GC content between 30% and 70%, and possessed no poly-U stretches longer than 4 nt. We then converted the target sequence into the reverse complement (red line) and entered this spacer sequence into mFold (condition: 25°C) for hairpin analysis. This was repeated until four optimal target sites were selected. **(B)** Generation of gRNA^{array} construct. Double-stranded DNA was first synthesized to contain four spacer and five DR sequences with specific restriction sites present on the 5′ and 3′ end of the DNA. Simultaneously, the vector backbone containing the *miniwhite* marker, a U6:3 promoter fragment, and an attB site was digested using the corresponding restriction sites of the dsDNA gene fragment. The two pieces were then ligated together to generate a CasRx gRNA^{array} covering the majority of the transcript for the GOI. CasRx, Cas ribonuclease; gRNA^{array}, guide RNA array.
