# Supplementary material for: Programmable RNA Targeting Using CasRx in Flies
Source: CRISPR J. 2020 Jun 17;3(3):164–76. doi: 10.1089/crispr.2020.0018 (PMC7307691; doi:10.1089/crispr.2020.0018)
Supplement: Supplemental data [file Supp_FigS2.pdf]

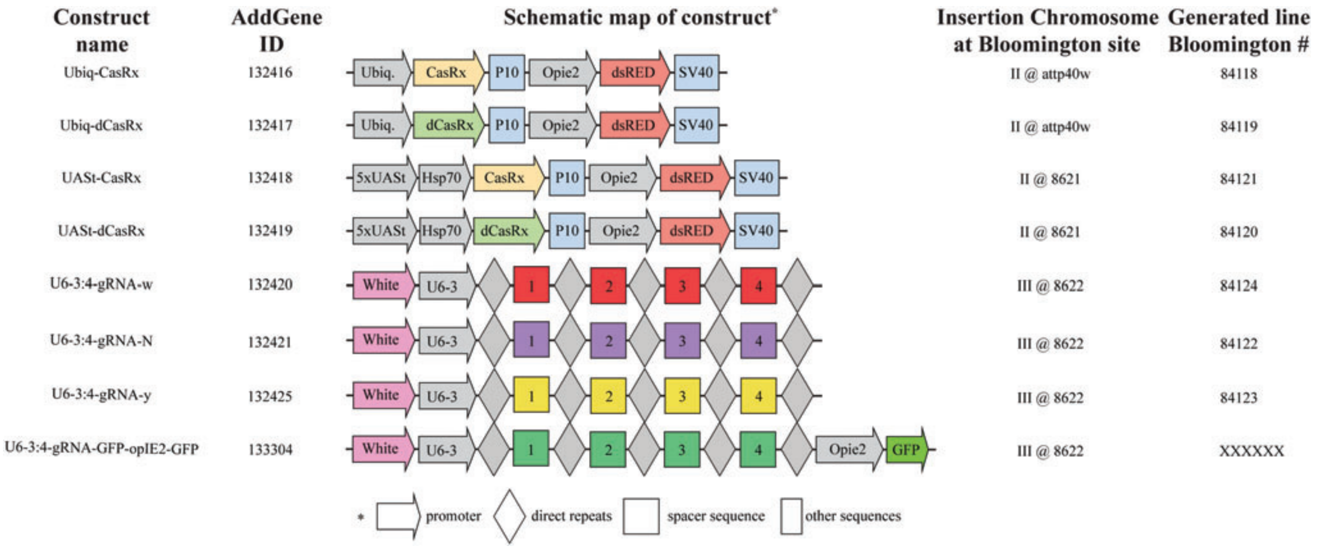

Supplementary Fig. S2. Schematic representation of constructs generated for this study. All constructs used in this study are depicted here along with addgene ID, insertion site, and Bloomington stock number.
