# Supplementary material for: Programmable RNA Targeting Using CasRx in Flies
Source: CRISPR J. 2020 Jun 17;3(3):164–76. doi: 10.1089/crispr.2020.0018 (PMC7307691; doi:10.1089/crispr.2020.0018)
Supplement: Supplemental data [file Supp_FigS3.pdf]

Development Stage	Expression Level notch	white	yellow
embryo 00-02hr	44	0	0
embryo 02-04hr	49	0	1
embryo 04-06hr	86	2	0
embryo 06-08hr	124	3	0
embryo 08-10hr	102	4	0
embryo 10-12hr	52	9	0
embryo 12-14hr	53	8	0
embryo 14-16hr	41	9	2
embryo 16-18hr	19	9	26
embryo 18-20hr	10	7	28
embryo 20-22hr	10	4	19
embryo 22-24hr	10	4	21
larva L1	6	9	3
larva L2	4	9	11
larva L3 12hr	4	9	9
larva L3 puffstage 1-2	7	8	0
larva L3 puffstage 3-6	21	13	0
larva L3 puffstage 7-9	23	21	0
white prepupa	29	30	0
prepupa 12hr	30	10	1
pupa 1d	52	9	0
pupa 2d	21	24	26
pupa 3d	9	12	47
pupa 4d	6	6	2
adult male 01 day	4	11	2
adult male 05 day	3	11	0
adult male 30 day	3	11	0
adult female 01 day	14	7	1
adult female 05 day	13	3	0
adult female 30 day	15	4	0

- no expression (0-0)
- Very low expression (1-3)
- Low expression (4-10)
- Moderate Expression (11-25)
- Moderately High expression (26-50)
- High expression (51-100)
- Very high expression (101-1000)
- Extremely high expression (>1000)

Supplementary Fig. S3. modENCODE transcript expression relative to *Drosophila melanogaster* development. Black box indicates which developmental period was chosen for RNA sequencing of samples for the analysis of CasRx-mediated transcript reduction in Ubiq-CasRx versus Ubiq-dCasRx comparison. Not included: GFP first instar larvae were chosen for analysis of *GFP* transcript reduction. GFP, green fluorescent protein.
