# Supplementary material for: Programmable RNA Targeting Using CasRx in Flies
Source: CRISPR J. 2020 Jun 17;3(3):164–76. doi: 10.1089/crispr.2020.0018 (PMC7307691; doi:10.1089/crispr.2020.0018)
Supplement: Supplemental data [file Supp_FileS1.pdf]

```perl
#!/usr/bin/perl -w

use strict;

if ($#ARGV != 0) {
    die "usage: $0 counts\n";
}

open (IN, "<$ARGV[0]") || die $!;

my @data=();
my $rate_sum=0;
my $count_sum=0;
while (<IN>) {
    if (/^#/) {
            print;
            next;
    }
    chomp;
    my @tmp=split(/\t/);
    if ($tmp[0] eq 'Geneid') {
            $tmp[6]='Count';
            print join("\t", @tmp), "\tTPM\tFPKM\n";
#           print "$_\tTPM\tFPKM\n";
            next;
    }
    my $rpk=1000*$tmp[6]/$tmp[5]; #RPK
    $rate_sum+=$rpk;
    $count_sum+=$tmp[6];
#    push(@tmp, $rate);
    push (@data, [@tmp]);
}
#print "rate_sum=$rate_sum\n";

foreach (@data) {
    my $rpk=1000*${$_}[6]/${$_}[5];
    my $tpm=1000000*$rpk/$rate_sum;
    my $fpm=1000000*${$_}[6]/$count_sum;
    my $fpkm=1000*$fpm/${$_}[5];
    print join("\t", @{$_}), "\t$tpm\t$fpkm\n";
}
```
