# Supplementary material for: Programmable RNA Targeting Using CasRx in Flies
Source: CRISPR J. 2020 Jun 17;3(3):164–76. doi: 10.1089/crispr.2020.0018 (PMC7307691; doi:10.1089/crispr.2020.0018)
Supplement: Supplemental data [file Supp_FileS2.pdf]

```
setwd("D:/Akbari/CasRx") # Please change this to your working directory
where the raw count data is stored.
library(magrittr)
library(tidyverse)
library(dplyr)
library(ggplot2)
library(reshape2)
library(DESeq2)

## RNA-seq results

countData <- read.csv("CasRx-selected-RNAseq-Igor/countData.csv", header
= T, stringsAsFactors = F)

# Volcano plots

########## GFP
GFP_Data <- as.matrix(countData[, c(6, 7, 18, 19, 30, 31)])
for (i in 1:6){
  for (j in 1:nrow(countData)){
    GFP_Data[j, i] = as.integer(GFP_Data[j, i])
  }
}
condition <- factor(c("CasRx", "dCasRx", "CasRx", "dCasRx", "CasRx",
"dCasRx"))
dds <- DESeqDataSetFromMatrix(GFP_Data, DataFrame(condition), ~
condition)
dds <- DESeq(dds)
res <- results(dds)
resultsNames(dds)
resLFC <- lfcShrink(dds, coef=2)
resLFC[which(countData[,1] == "GFP"),]

GFP_df <- data.frame(countData$ID, resLFC$baseMean,
resLFC$log2FoldChange, resLFC$padj)
colnames(GFP_df) <- c("ID", "baseMean", "log2FoldChange", "padj")

temp_df = GFP_df
temp_df$padj_neglog = -log(temp_df$padj)
p <- ggplot() +
  scale_y_continuous(trans='log10',  limits = c(0.00001, 1000))+
  geom_point(data = temp_df[(temp_df$padj> 0.05), ],aes(x =
log2FoldChange, y = padj_neglog), size = 1.5, col = 'grey50') +
  geom_point(data = temp_df[(temp_df$padj< 0.05), ],aes(x =
log2FoldChange, y = padj_neglog), size = 1.5, col = 'brown4') +
  geom_point(data = temp_df[temp_df$ID == "GFP", ],aes(x =
log2FoldChange, y = padj_neglog), size = 1.5, col = 'hotpink', shape = 0,
stroke = 2) +
  theme_minimal() +
  theme(panel.border = element_blank(),
        panel.grid.major = element_blank(),
        panel.grid.minor = element_blank(),
        axis.line = element_blank(),
        axis.text.x = element_text(size = 15),
```

```
        axis.text.y = element_text(angle = 0, size = 15),
        axis.title.y = element_text(size = 20),
        axis.title.x = element_text(size = 20)) +
  ylab(bquote('- ' *log[10]* 'adjusted P value')) +
  xlab(bquote('MAP ' *log[2]* 'Fold Change'))+
  xlim(-10,10)
ggsave(plot=p,height=6,width=8,dpi=200, filename="GFP_volcano.pdf",
useDingbats=FALSE)

######### w
w_Data <- as.matrix(countData[, c(2, 3, 14, 15, 26, 27)])
for (i in 1:6){
  for (j in 1:nrow(countData)){
    w_Data[j, i] = as.integer(w_Data[j, i])
  }
}
condition <- factor(c("CasRx", "dCasRx", "CasRx", "dCasRx", "CasRx",
"dCasRx"))
dds_w <- DESeqDataSetFromMatrix(w_Data, DataFrame(condition), ~
condition)
dds_w <- DESeq(dds_w)
res_w <- results(dds_w)
resultsNames(dds_w)
resLFC_w <- lfcShrink(dds_w, coef=2)
resLFC_w[which(countData[,1] == "FBgn0003996"),]

w_df <- data.frame(countData$ID, resLFC_w$baseMean,
resLFC_w$log2FoldChange, resLFC_w$padj)
colnames(w_df) <- c("ID", "baseMean", "log2FoldChange", "padj")

temp_df = w_df
temp_df$padj_neglog = -log(temp_df$padj)
p <- ggplot() +
  scale_y_continuous(trans='log10', limits = c(0.00001, 1000))+
  geom_point(data = temp_df[(temp_df$padj> 0.05), ],aes(x =
log2FoldChange, y = padj_neglog), size = 1.5, col = 'grey50') +
  geom_point(data = temp_df[(temp_df$padj< 0.05), ],aes(x =
log2FoldChange, y = padj_neglog), size = 1.5, col = 'brown4') +
  geom_point(data = temp_df[temp_df$ID == "FBgn0003996", ],aes(x =
log2FoldChange, y = padj_neglog), size = 1.5, col = 'hotpink', shape = 0,
stroke = 2) +
  theme_minimal() +
  theme(panel.border = element_blank(),
        panel.grid.major = element_blank(),
        panel.grid.minor = element_blank(),
        axis.line = element_blank(),
        axis.text.x = element_text(size = 15),
        axis.text.y = element_text(angle = 0, size = 15),
        axis.title.y = element_text(size = 20),
        axis.title.x = element_text(size = 20)) +
  ylab(bquote('- ' *log[10]* 'adjusted P value')) +
  xlab(bquote('MAP ' *log[2]* 'Fold Change'))+
  xlim(-10,10)
```

```
ggsave(plot=p,height=6,width=8,dpi=200, filename="w_volcano.pdf",
useDingbats=FALSE)

########## N

N_Data <- as.matrix(countData[, c(4, 5, 16, 17, 28, 29)])
for (i in 1:6){
  for (j in 1:nrow(countData)){
    N_Data[j, i] = as.integer(N_Data[j, i])
  }
}
condition <- factor(c("CasRx", "dCasRx", "CasRx", "dCasRx", "CasRx",
"dCasRx"))
dds_N <- DESeqDataSetFromMatrix(N_Data, DataFrame(condition), ~
condition)
dds_N <- DESeq(dds_N)
res_N <- results(dds_N)
resultsNames(dds_N)
resLFC_N <- lfcShrink(dds_N, coef=2)
resLFC_N[which(countData[,1] == "FBgn0004647"),]

N_df <- data.frame(countData$ID, resLFC_N$baseMean,
resLFC_N$log2FoldChange, resLFC_N$padj)
colnames(N_df) <- c("ID", "baseMean", "log2FoldChange", "padj")

temp_df = N_df
temp_df$padj_neglog = -log(temp_df$padj)
p <- ggplot() +
  scale_y_continuous(trans='log10', limits = c(0.00001, 1000))+
  geom_point(data = temp_df[(temp_df$padj> 0.05), ],aes(x =
log2FoldChange, y = padj_neglog), size = 1.5, col = 'grey50') +
  geom_point(data = temp_df[(temp_df$padj< 0.05), ],aes(x =
log2FoldChange, y = padj_neglog), size = 1.5, col = 'brown4') +
  geom_point(data = temp_df[temp_df$ID == "FBgn0004647", ],aes(x =
log2FoldChange, y = padj_neglog), size = 1.5, col = 'hotpink', shape = 0,
stroke = 2) +
  theme_minimal() +
  theme(panel.border = element_blank(),
        panel.grid.major = element_blank(),
        panel.grid.minor = element_blank(),
        axis.line = element_blank(),
        axis.text.x = element_text(size = 15),
        axis.text.y = element_text(angle = 0, size = 15),
        axis.title.y = element_text(size = 20),
        axis.title.x = element_text(size = 20)) +
  ylab(bquote('- ' *log[10]* 'adjusted P value')) +
  xlab(bquote('MAP ' *log[2]* 'Fold Change'))+
  xlim(-10,10)
ggsave(plot=p,height=6,width=8,dpi=200, filename="N_volcano.pdf",
useDingbats=FALSE)
```

```
########### y

y_Data <- as.matrix(countData[, c(12, 13, 24, 25, 36, 37)])
for (i in 1:6){
  for (j in 1:nrow(countData)){
    y_Data[j, i] = as.integer(y_Data[j, i])
  }
}
condition <- factor(c("CasRx", "dCasRx", "CasRx", "dCasRx", "CasRx",
"dCasRx"))
dds_y <- DESeqDataSetFromMatrix(y_Data, DataFrame(condition), ~
condition)
dds_y <- DESeq(dds_y)
res_y <- results(dds_y)
resultsNames(dds_y)
resLFC_y <- lfcShrink(dds_y, coef=2)
resLFC_y[which(countData[,1] == "FBgn0004034"),]

y_df <- data.frame(countData$ID, resLFC_y$baseMean,
resLFC_y$log2FoldChange, resLFC_y$padj)
colnames(y_df) <- c("ID", "baseMean", "log2FoldChange", "padj")

temp_df = y_df
temp_df$padj_neglog = -log(temp_df$padj)
p <- ggplot() +
  scale_y_continuous(trans='log10', limits = c(0.00001, 1000))+
  geom_point(data = temp_df[(temp_df$padj> 0.05), ],aes(x =
log2FoldChange, y = padj_neglog), size = 1.5, col = 'grey50') +
  geom_point(data = temp_df[(temp_df$padj< 0.05), ],aes(x =
log2FoldChange, y = padj_neglog), size = 1.5, col = 'brown4') +
  geom_point(data = temp_df[temp_df$ID == "FBgn0004034", ],aes(x =
log2FoldChange, y = padj_neglog), size = 1.5, col = 'hotpink', shape = 0,
stroke = 2) +
  theme_minimal() +
  theme(panel.border = element_blank(),
        panel.grid.major = element_blank(),
        panel.grid.minor = element_blank(),
        axis.line = element_blank(),
        axis.text.x = element_text(size = 15),
        axis.text.y = element_text(angle = 0, size = 15),
        axis.title.y = element_text(size = 20),
        axis.title.x = element_text(size = 20)) +
  ylab(bquote('- ' *log[10]* 'adjusted P value')) +
  xlab(bquote('MAP ' *log[2]* 'Fold Change'))+
  xlim(-10,10)
ggsave(plot=p,height=6,width=8,dpi=200, filename="y_volcano.pdf",
useDingbats=FALSE)
```
