# Supplementary material for: Programmable RNA Targeting Using CasRx in Flies
Source: CRISPR J. 2020 Jun 17;3(3):164–76. doi: 10.1089/crispr.2020.0018 (PMC7307691; doi:10.1089/crispr.2020.0018)
Supplement: Supplemental data [file Supp_FigS4.pdf]

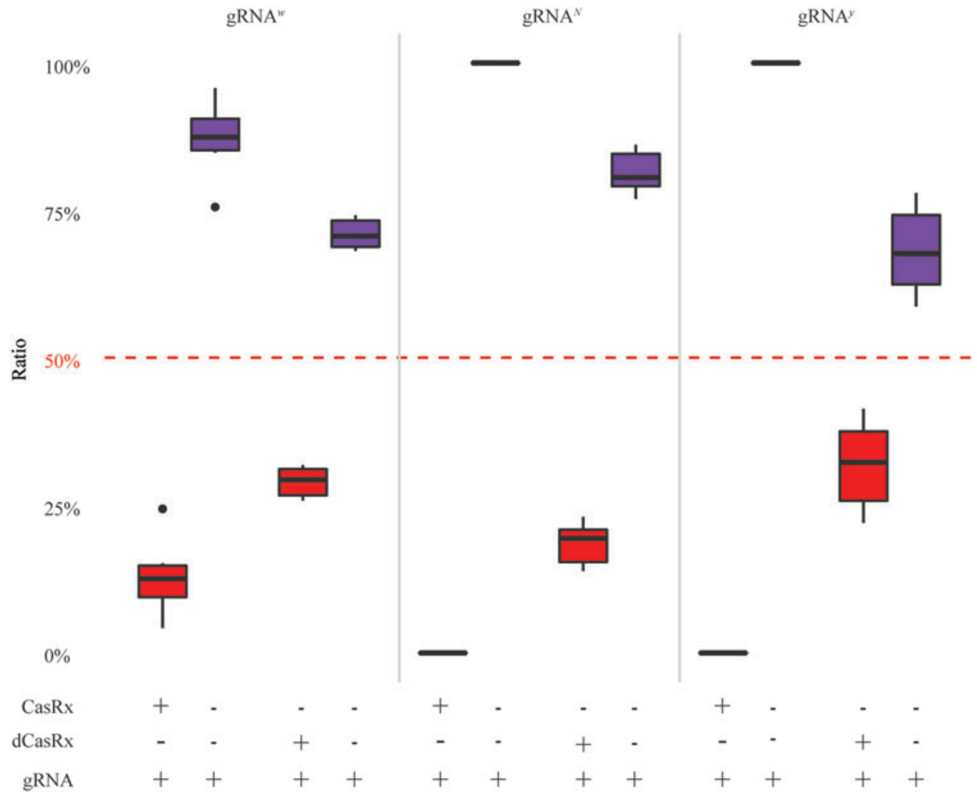

Supplementary Fig. S4. Complete inheritance data for bidirectional crosses. Complete inheritance plot for the bidirectional crosses featured in Fig 2. The plot includes all genotypes scored in all crosses between either Ubiq-CasRx or Ubiq-dCasRx and a respective gRNA^{array}. In all crosses, gRNA^{array}-only inheritance is dramatically higher than transheterozygote inheritance rates, including for Ubiq-dCasRx crosses.
