# Supplementary material for: Programmable RNA Targeting Using CasRx in Flies
Source: CRISPR J. 2020 Jun 17;3(3):164–76. doi: 10.1089/crispr.2020.0018 (PMC7307691; doi:10.1089/crispr.2020.0018)
Supplement: Supplemental data [file Supp_FigS5.pdf]

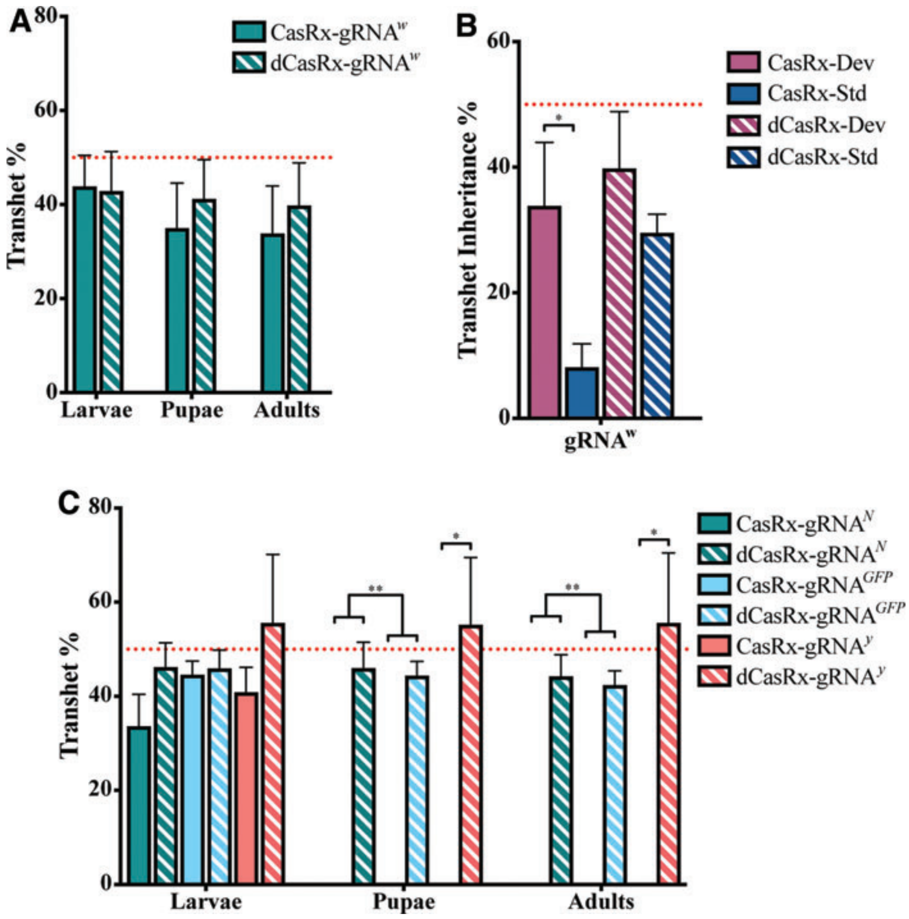

Supplementary Fig. S5. Development-related inheritance and lethality of Ubiq-CasRx and Ubiq-dCasRx transheterozygotes. **(A–B)** Transheterozygote percentages at larval, pupal, and adult developmental periods for each gRNA^{array} that produced an observable phenotype (*w*). There were no significant differences in inheritance. **(C)** Transheterozygote percentages through larval, pupal, and adult development periods for each gRNA^{array} that produced a lethal phenotype (*N*, *y*, *GFP*). No Ubiq-CasRx transheterozygotes developed beyond larvae. *N*, *Notch* gene; *w*, *white* gene; *y*, *yellow* gene.
