# Supplementary material for: Programmable RNA Targeting Using CasRx in Flies
Source: CRISPR J. 2020 Jun 17;3(3):164–76. doi: 10.1089/crispr.2020.0018 (PMC7307691; doi:10.1089/crispr.2020.0018)
Supplement: Supplemental data [file Supp_FigS6.pdf]

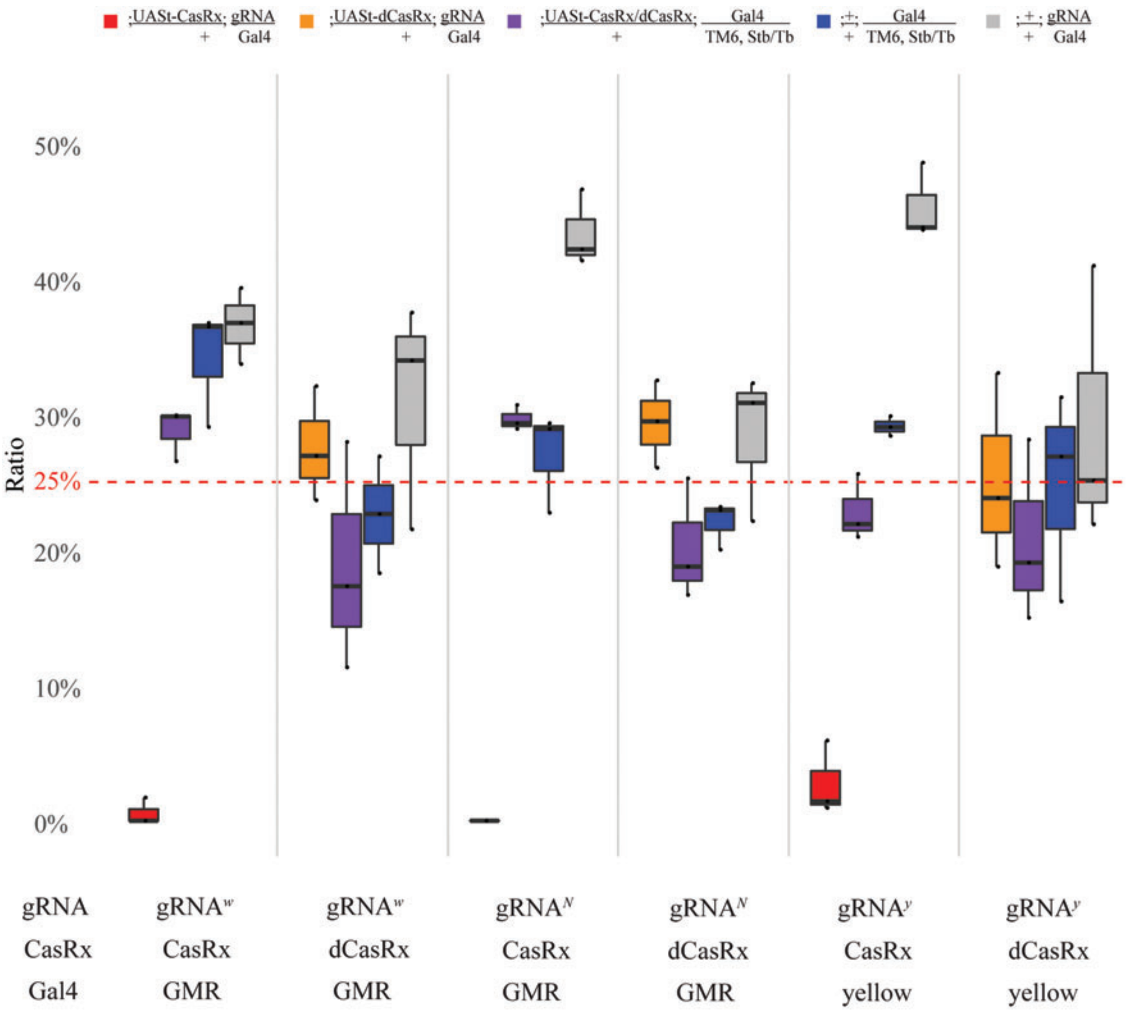

Supplementary Fig. S6. Complete inheritance data for binary Gal4/UAS crosses. The plot includes all genotypes scored in all crosses for UASt-CasRx and UASt-dCasRx. For all five gRNA^{array} targets, the inheritance of transheterozygous progeny expressing UASt-CasRx, a Gal4 driver, and a gRNA^{array} are lower compared with the other nontransheterozygous flies and compared with their corresponding dCasRx control group expressing UASt-dCasRx, a Gal4 driver, and a gRNA^{array}. dCasRx, catalytically inactive negative control.
